# Publisher Correction: Hypoxia-activated neuropeptide Y/Y5 receptor/RhoA pathway triggers chromosomal instability and bone metastasis in Ewing sarcoma

**DOI:** 10.1038/s41467-022-30473-7

**Published:** 2022-05-12

**Authors:** Congyi Lu, Akanksha Mahajan, Sung-Hyeok Hong, Susana Galli, Shiya Zhu, Jason U. Tilan, Nouran Abualsaud, Mina Adnani, Stacey Chung, Nada Elmansy, Jasmine Rodgers, Olga Rodriguez, Christopher Albanese, Hongkun Wang, Maureen Regan, Valerie Zgonc, Jan Blancato, Ewa Krawczyk, G. Ian Gallicano, Michael Girgis, Amrita Cheema, Ewa Iżycka-Świeszewska, Luciane R. Cavalli, Svetlana D. Pack, Joanna Kitlinska

**Affiliations:** 1 Department of Physiology and Biophysics, Georgetown University, Washington, DC USA; 2 New York Genome Center, New York, NY USA; 3 Department of Biochemistry and Molecular & Cellular Biology, Georgetown University Medical Center, Washington, DC USA; 4 Department of Human Science, School of Nursing and Health Studies, Georgetown University, Washington, DC USA; 5 Cell Therapy and Cancer Research Department, King Abdullah International Medical Research Center, Riyadh, Saudi Arabia; 6 King Saud bin Abdulaziz University for Health Sciences, Riyadh, Saudi Arabia; 7 Department of Oncology, Lombardi Comprehensive Cancer Center, Georgetown University Medical Center, Washington, DC USA; 8 Center for Translational Imaging, Georgetown University Medical Center, Washington, DC USA; 9 Department of Biostatistics, Bioinformatics and Biomathematics, Georgetown University, Washington, DC USA; 10 Genome Editing Core, University of Illinois, Chicago, IL USA; 11 Laboratory of Pathology, National Cancer Institute, National Institutes of Health, Bethesda, MD USA; 12 Center for Cell Reprogramming, Georgetown University Medical Center, Washington, DC USA; 13 Department of Pathology and Neuropathology, Medical University of Gdańsk, Gdańsk, Poland; 14 Research Institute Pelé Pequeno Príncipe, Faculdades Pequeno Príncipe, Curitiba, PR Brazil

**Keywords:** Cancer microenvironment, Bone metastases, Paediatric cancer, Sarcoma

Correction to: *Nature Communications* 10.1038/s41467-022-29898-x, published online 28 April 2022.

In this article Sung-Hyeok Hong should have been denoted as an equally contributing author. The original article has been corrected.

