# Supplementary material for: Hypoxia-activated neuropeptide Y/Y5 receptor/RhoA pathway triggers chromosomal instability and bone metastasis in Ewing sarcoma
Source: Nat Commun. 2022 Apr 28;13:2323. doi: 10.1038/s41467-022-29898-x (PMC9051212; doi:10.1038/s41467-022-29898-x)
Supplement: Supplementary file 1 — Supplementary Information [file 41467_2022_29898_MOESM1_ESM.pdf]

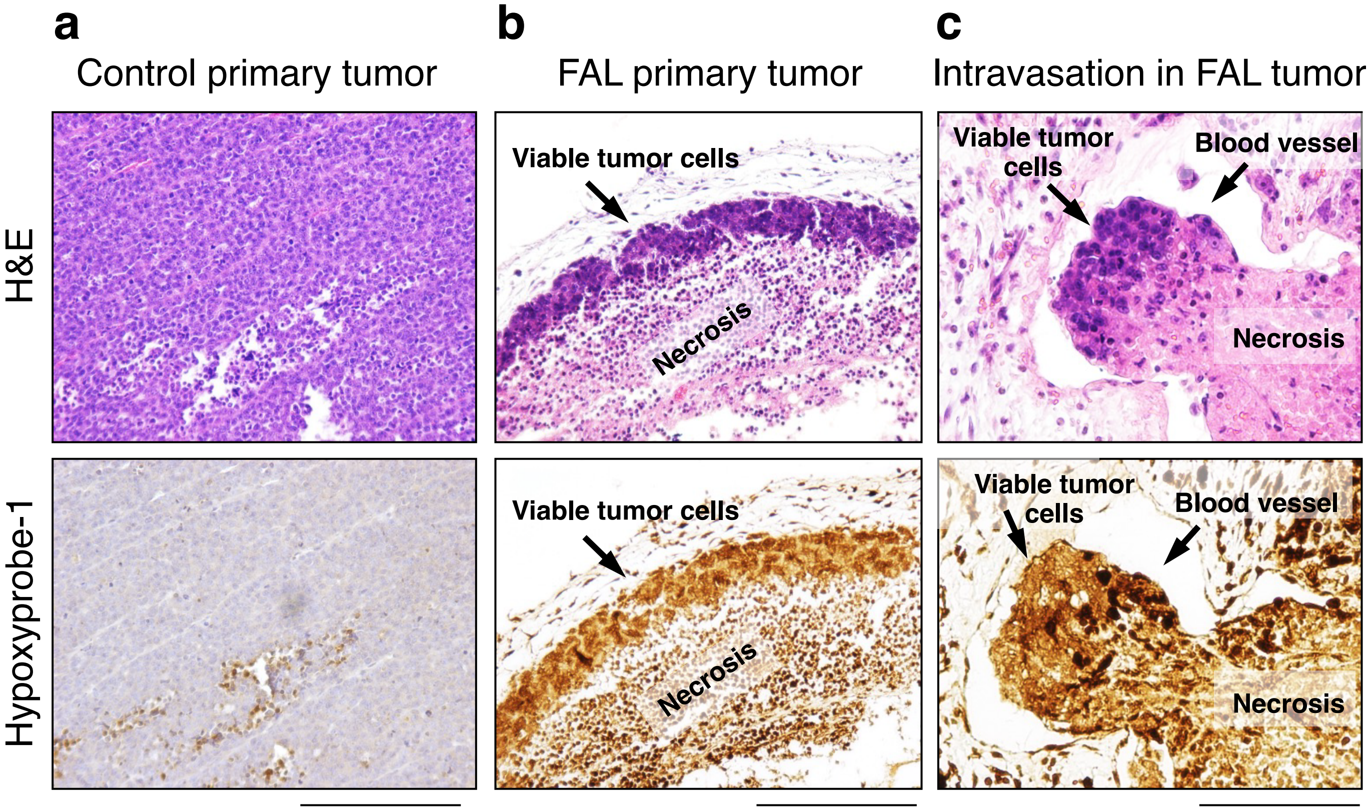

Supplementary figure 1. **Femoral artery ligation (FAL) triggers hypoxia in ES xenografts. a** Representative images of control SK-ES-1 tumors at a size of 150mm^3, stained with H&E and immunostained for hypoxyprobe-1 (HP-1) (n = 26). **b-c** Representative images of a similarly sized tumor from a FAL-treated mouse, stained with H&E and anti-HP-1 antibody under low (b) and high (c) magnification (n = 26). Scale bar: 200μm for (a) and (b), 100 μm for (c)

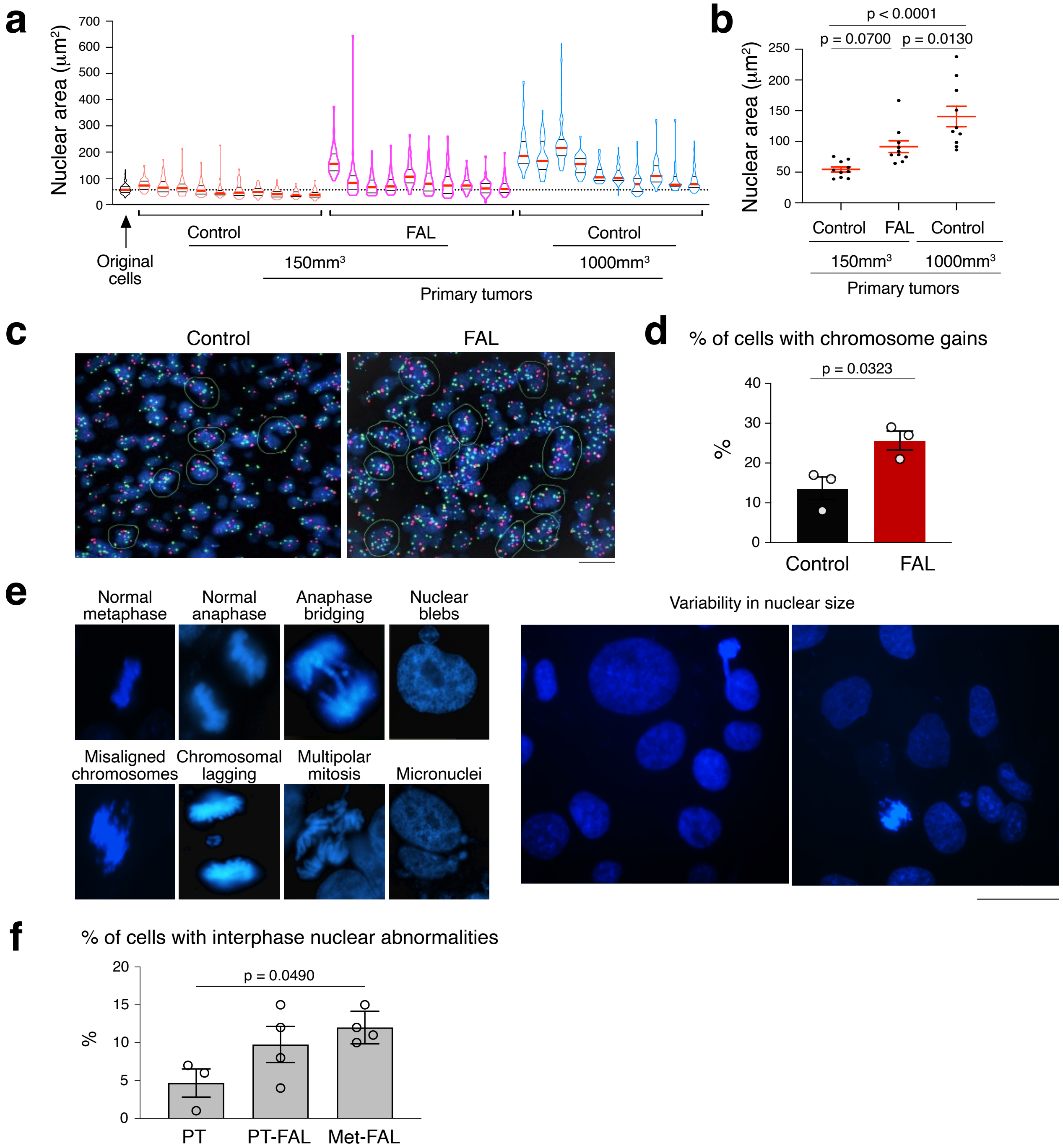

Supplementary figure 2. **Hypoxic primary tumors and their corresponding metastases exhibit signs of chromosomal instability. a** Violin plot shows the analysis of nuclear area of the original SK-ES-1 cells (n = 45) and cells isolated from individual SK-ES-1 xenografts from small (150 mm³) control (n = 28, 53, 45, 40, 36, 38, 44, 45, 43, 45) or FAL-treated tumors (n = 32, 41, 40, 41, 41, 47, 35, 41, 40, 47) or large (1000 mm³) untreated primary tumors (n = 26, 33, 63, 33, 46, 35, 53, 22, 18, 102). The numbers of cells examined are listed in the order presented in the graph. The red lines represent the median; the black lines represent the quartiles. **b** Analysis of mean nuclear size for the SK-ES-1 xenografts shown in panel a. One-way ANOVA followed by Tukey's test. **c** Representative images of FISH with CDKNA2/CEN3/7/17 probes in metastatic tissues from SK-ES-1 xenografts of control and FAL-treated mice (n = 3 per group). Scale bar: 10μm. **d** Analysis of percentage of cells with chromosome gains compared between metastases from control and FAL groups (n = 3 metastases per group). Two-tailed unpaired t-test. **e** Examples of mitotic segregation errors and nuclear abnormalities observed in cells isolated from bone metastases arising in FAL-treated mice (n = 78). Scale bar: 50μm. **f** Frequency of interphase nuclear abnormalities (micronuclei and nuclear blebs) in cells isolated from SK-ES-1 control primary tumors (PT) (n = 3), FAL-treated primary tumors (PT-FAL) (n = 4) or metastases from FAL-treated mice (Met-FAL) (n = 4). One-way ANOVA followed by Dunnett's test. Error bars indicate standard error of the mean.

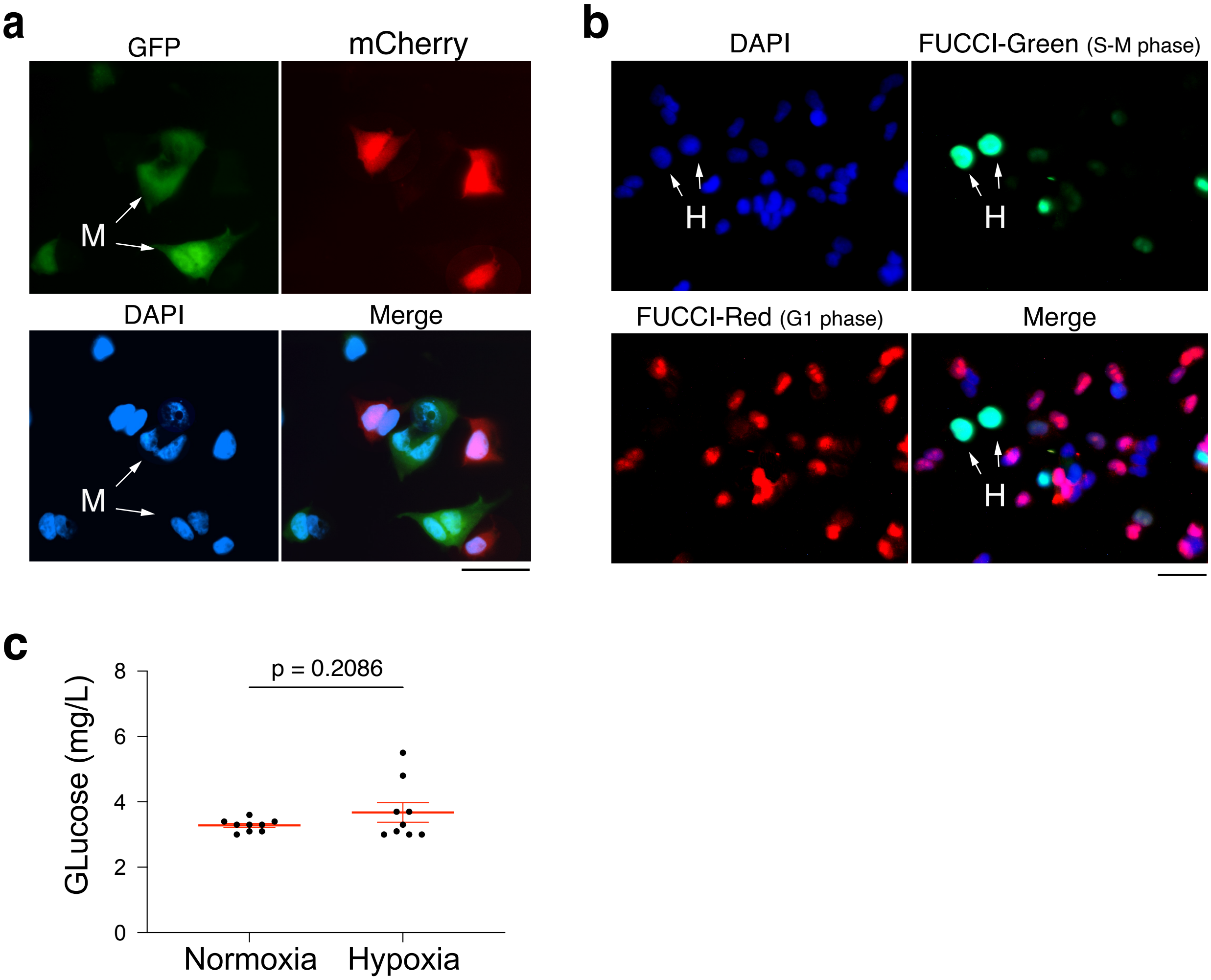

Supplementary figure 3. **Characteristics of ES cells exposed to hypoxia *in vitro*. a** Representative images of SK-ES-1 cells stably expressing GFP or mCherry and co-cultured in hypoxia (0.1% oxygen) for 24h. Multinucleated cells that developed under these conditions preserve expression of a single fluorescent protein (white arrows), excluding cell fusion as the mechanism of their formation (n = 2 independent experiments). Scale bar: 50μm. **b** Representative images of SK-ES-1 cells stably transfected with FUCCI cell cycle sensor and exposed to hypoxia for 72h. G1 phase marker, Cdt1-TagRFP, is expressed in the majority of cells, while the S-M phase marker, Geminin-GFP, is preferentially expressed in hypertrophic cells (white arrows) (n = 3 independent experiments). Scale bar: 50μm. **c** Analysis of glucose levels in conditioned media derived from SK-ES-1 cells cultured for 72h in normoxia or hypoxia (n = 9 per group). Two-tailed unpaired t-test. Error bars indicate standard error of the mean.

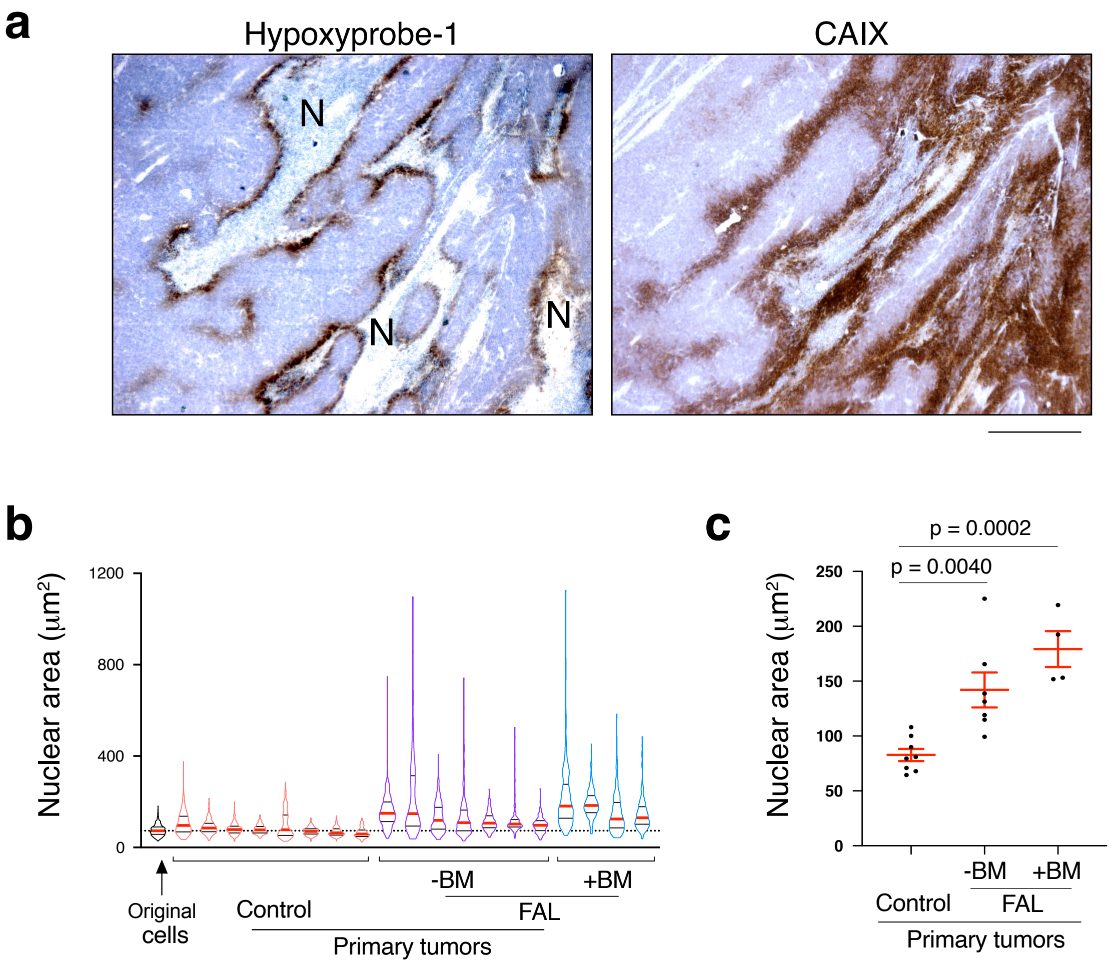

Supplementary figure 4. **Characteristics of ES primary tumors. a** Representative images of a large (1000 mm^3) TC32 primary tumor immunostained for an endogenous hypoxia marker, carbonic anhydrase IX (CAIX), and an exogenous hypoxyprobe-1 (HP-1) (n = 5). N - necrosis. Scale bar: 200μm. **b** Violin plot shows the analysis of nuclear area of the original TC71 cells (n = 285) and cells isolated from individual TC71 primary tumors from control (n = 237, 251, 148, 156, 314, 163, 137, 182) or FAL experimental groups. The FAL group was further divided into primary tumors that did (+BM) or did not (-BM) metastasize to bone (-BM n = 222, 134, 263, 205, 222, 172, 243; +BM n = 229, 244, 126, 113). The numbers of cells examined are listed in the order presented in the graph. The red lines represent the median; the black lines represent the quartiles. **c** Analysis of mean nuclear area for the TC71 xenografts shown in panel b (Control n = 8, FAL –BM n = 7, FAL +BM n = 4). One-way ANOVA followed by Dunnett's test. Error bars indicate standard error of the mean.

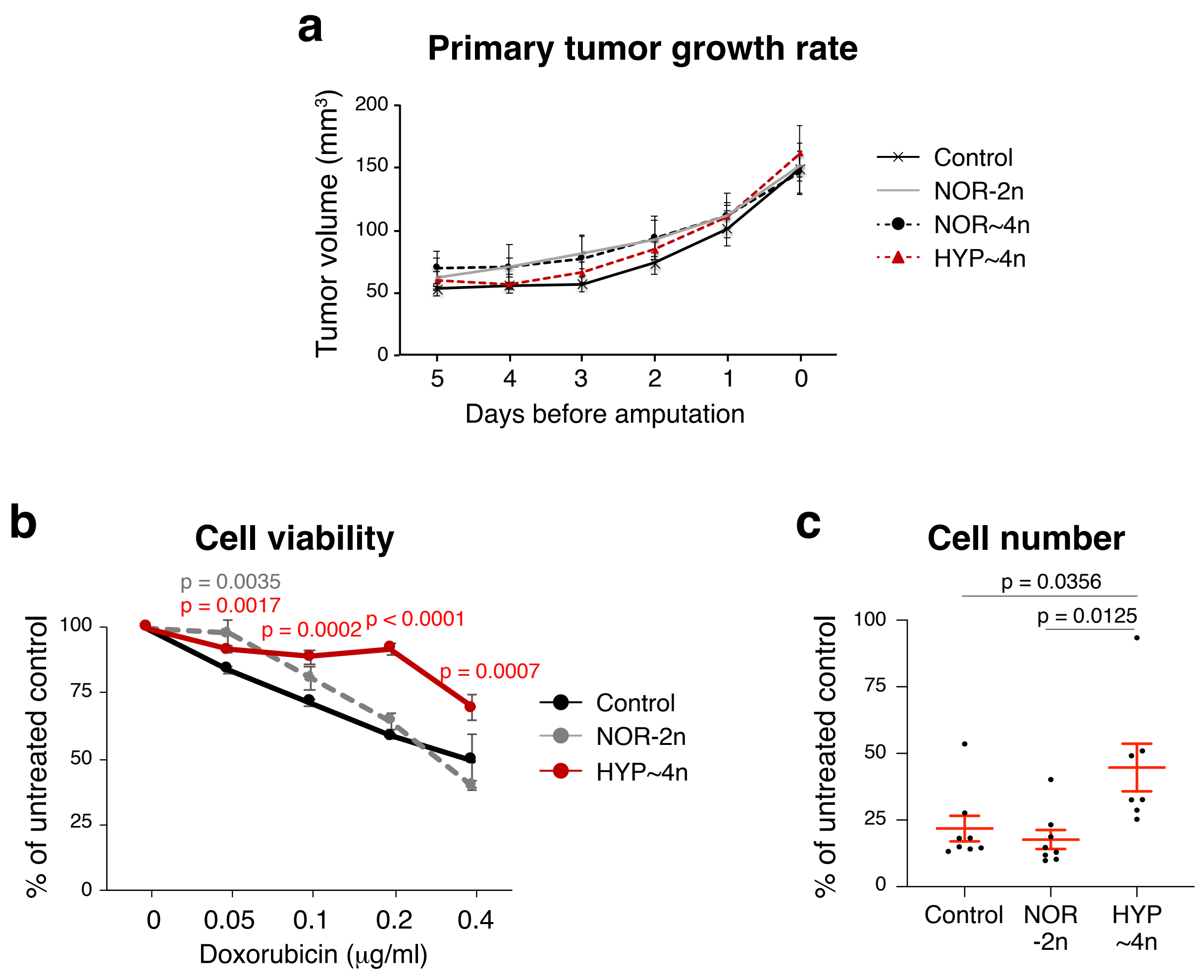

Supplementary figure 5. **Characteristics of SK-ES-1 2n and 4n cell progeny. a** Growth curve of SK-ES-1 xenografts arising from the original cells (Control) and the progeny of 2n and 4n cells isolated from normoxic (NOR) or hypoxic (HYP) cell cultures (Control n = 10, NOR-2n n = 4, NOR~4n n = 3, HYP~4n n = 7). **b** Viability of SK-ES-1 cell populations - unsorted (Control), diploid (NOR-2n) and the progeny of hypoxia-induced tetraploid cells (HYP~4n) – upon 72h treatment with doxorubicin, measured by MTS assay (n = 6 per group). Two-tailed unpaired t-test vs control. **c** Number of viable cells in the above cell types treated for 72h with doxorubicin (0.4 µg/ml; n = 4 independent experiments). One-way ANOVA followed by Tukey's test. Error bars indicate standard error of the mean.

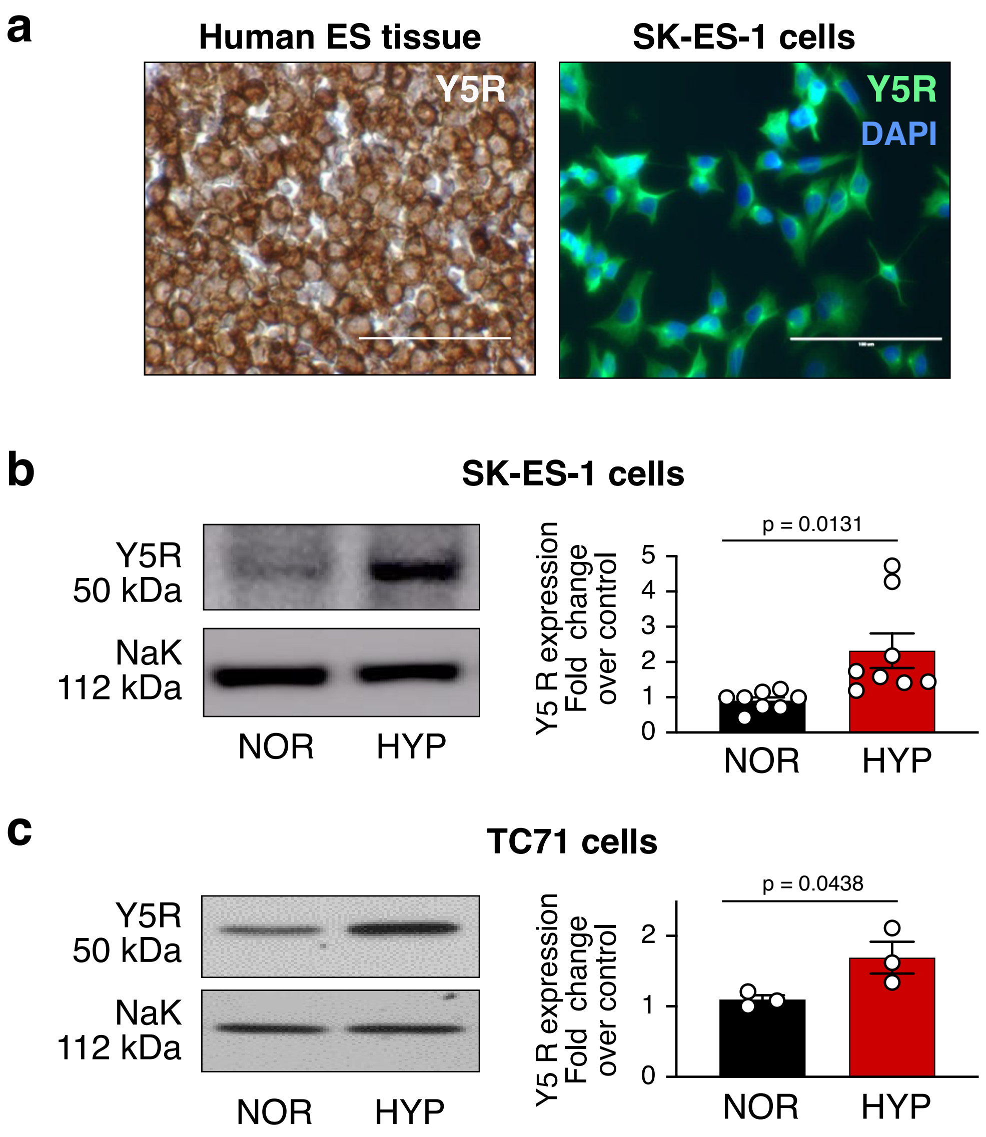

Supplementary figure 6. **The expression of Y5R in ES cells. a** Representative images of ES tissue and cells immunostained for Y5R (n = 15 for Human ES tissue, n = 3 for SK-ES-1 cells). Scale bar: 100μm. **b-c** Western blot analysis of Y5R expression in membrane protein fractions from normoxic and hypoxic SK-ES-1 (b) and TC71 (c) cells normalized to alpha 1 sodium potassium ATPase (NaK). The results of 3 independent experiments were quantified by densitometry and are presented here. Two-tailed paired t-test. Error bars indicate standard error of the mean.

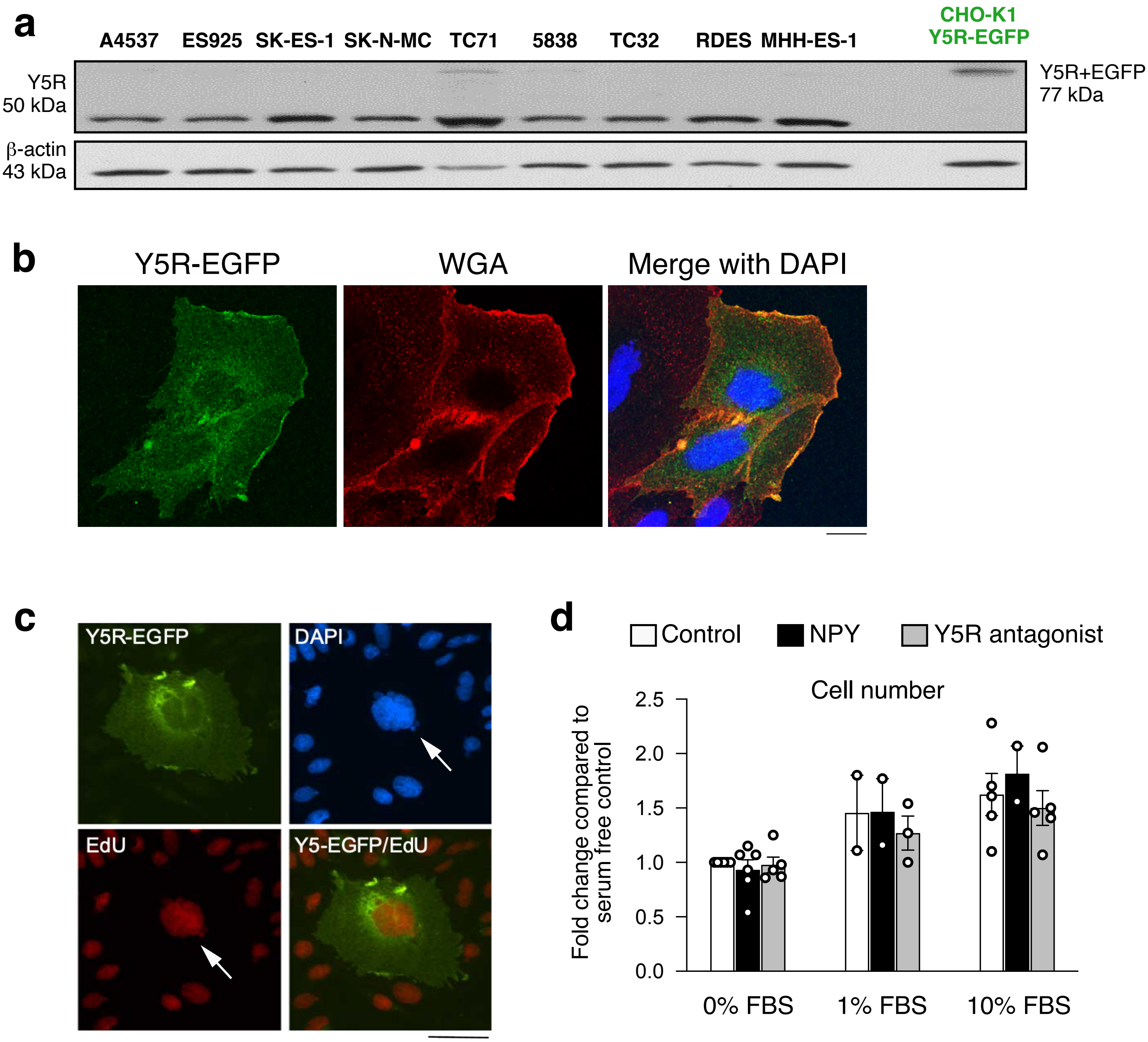

Supplementary figure 7. **Characteristics of CHO-K1/Y5R-EGFP transfectants. a** Protein levels of Y5R in whole cell extracts from a panel of ES cell lines and CHO-K1/Y5R-EGFP stable transfectants (n = 2 independent experiments). **b** Co-localization of Y5R-EGFP fusion protein with a cell membrane marker, wheat germ agglutinin (WGA, red) (n = 2 independent experiments). Scale bar: 50µm. **c** EdU uptake in CHO-K1 cells transiently transfected with Y5R-EGFP. White arrow - cell with a hypertrophic nucleus (n = 3 independent experiments). Scale bar: 100µm. **d** Effect of NPY (10^{-7}M) and Y5R antagonist (10^{-6}M) on cell number in CHO-K1/Y5R-EGFP stable transfectants. Cells were treated for 48h (n = 6, 3 and 5 independent experiments for 0%, 1% and 10% FBS, respectively). Error bars indicate standard error of the mean.

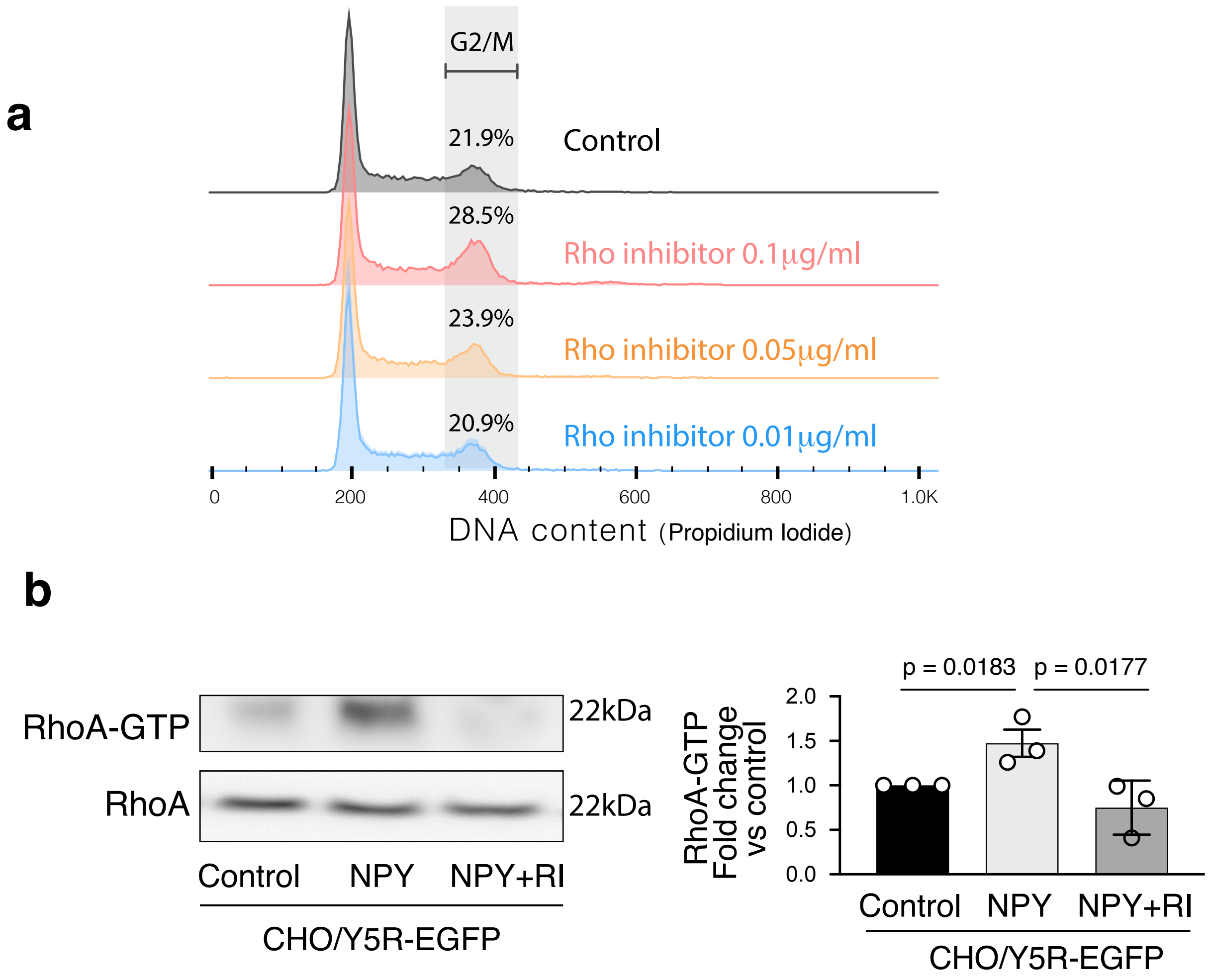

Supplementary figure 8. **Effectiveness of the Rho inhibitor I in CHO-K1/Y5R-EGFP cells.** **a** Flow cytometry analysis of DNA content in CHO-K1 cells treated for 2h with Rho inhibitor I at concentrations 0.01-0.1μg/ml. Rho inhibitor I at a concentration of 0.01 μg/ml did not cause a G2/M phase block and was used for all further analyses. **b** RhoA-GTP pull-down assay in CHO-K1/Y5R-EGFP cells treated with NPY (10^{-7}M) for 20min, with or without 2h pre-treatment with Rho inhibitor I at a concentration 0.01μg/ml. The results of 3 independent experiments were quantified by densitometry and are presented here. Two-sided unpaired t-test. Error bars indicate standard error of the mean.

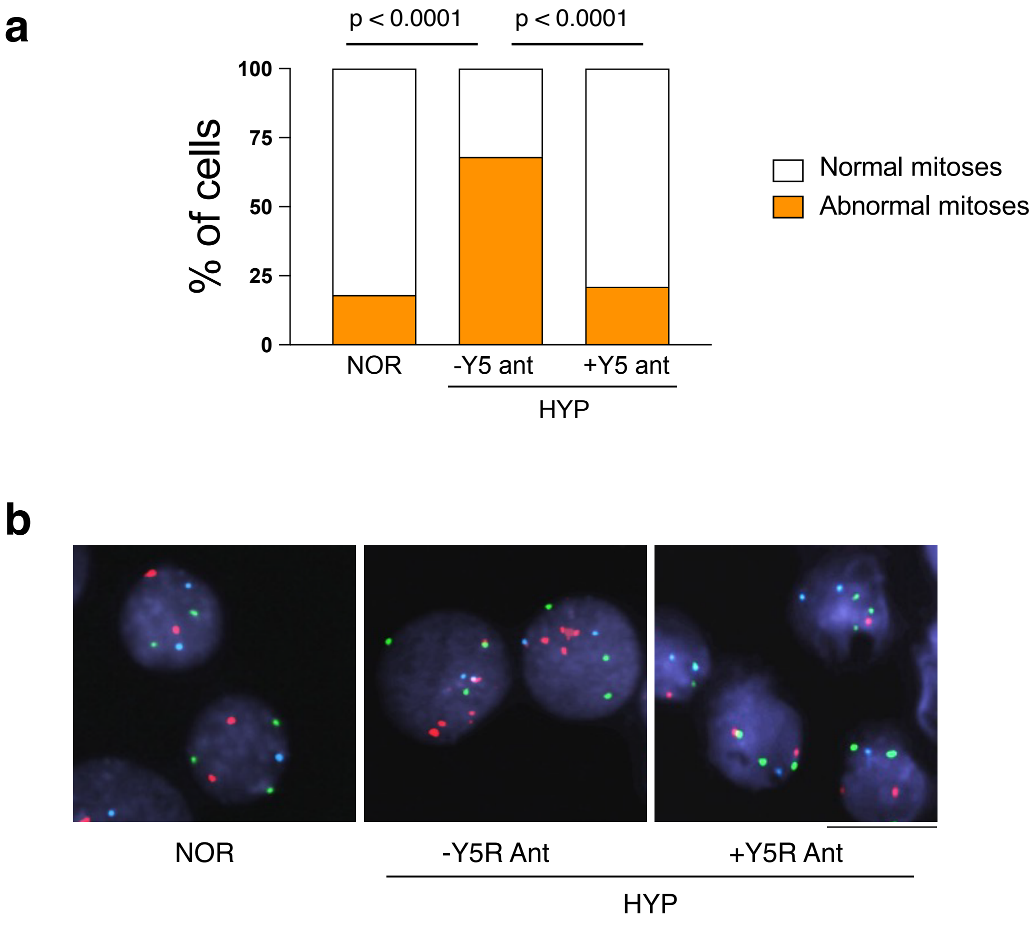

Supplementary figure 9. **Blocking Y5R prevents hypoxia-induced chromosomal instability and aneuploidy** **a** Analysis of mitotic segregation errors in SK-ES-1 cells subjected to hypoxia (HYP) for 72h followed by 24h culture in NOR, with or without Y5R antagonist (10^{-6}M) (NOR n = 66, HYP –Y5R Ant. n = 69, and HYP +Y5R Ant. n = 47 cells). Two-sided Fisher's exact test. **b** Representative images of FISH with CDKNA2/CEN3/7/17 probes in SK-ES-1 cells cultured in NOR or HYP for 72h, with or without Y5R antagonist (10^{-6}M) (NOR n = 137, HYP –Y5R Ant. and HYP +Y5R Ant. n = 110 cells). Scale bar: 10 μm.

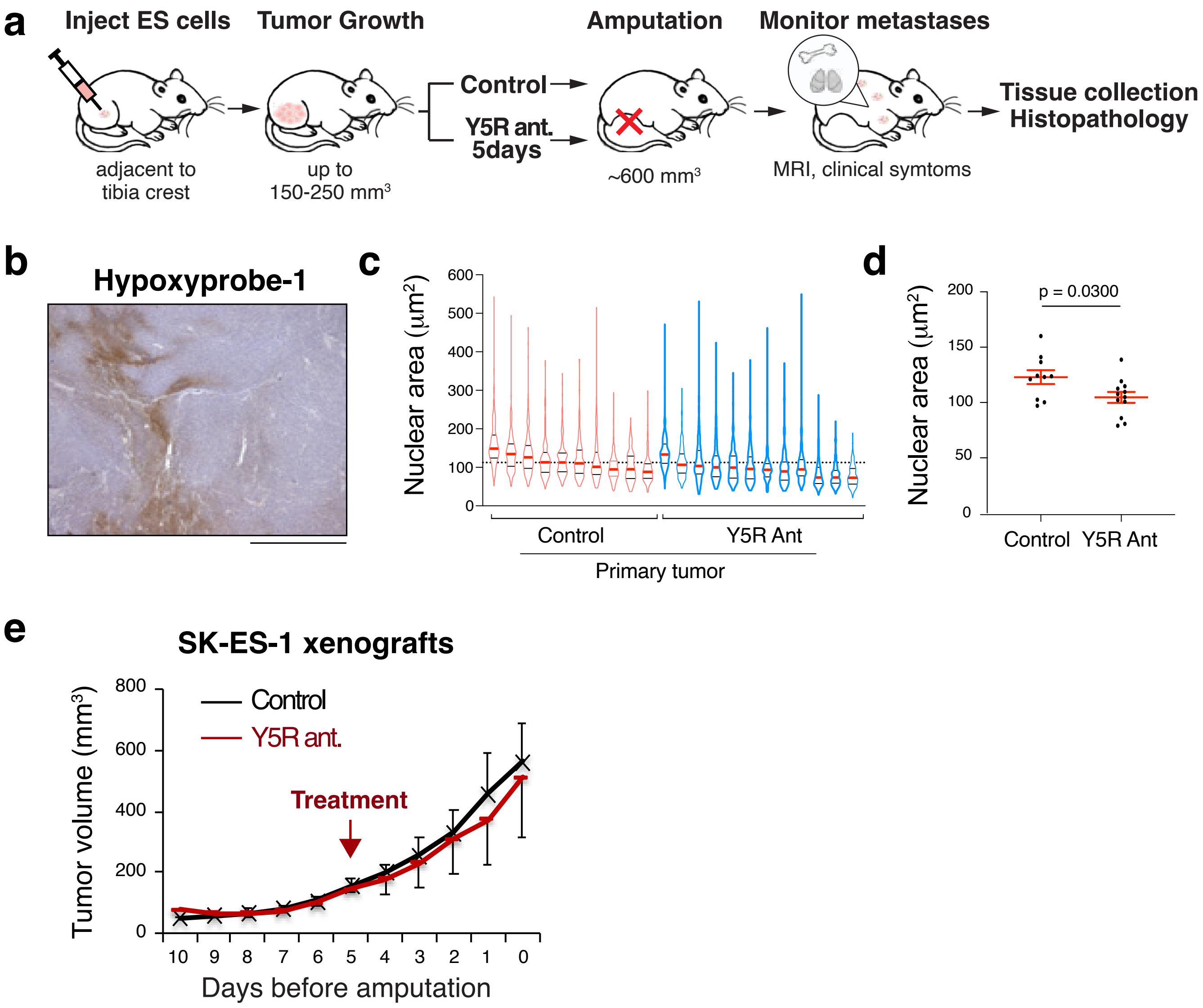

Supplementary figure 10. **Y5R antagonist treatment *in vivo.*** **a** Schematic representation of experimental procedures for testing the effect of Y5R antagonist (Y5R Ant) on ES metastasis developing during the period of increasing tumor hypoxia. **b** Representative image of hypoxic regions in SK-ES-1 xenografts at the 600 mm^3 volume. Hypoxic cells were detected by immunostaining for hypoxyprobe-1 (n = 4). Scale bar: 100µm. **c** Violin plot shows the analysis of nuclear area of cells isolated from individual SK-ES-1 primary tumors from control or Y5R antagonist-treated (CGP 71683, 20mg/kg, 5 days) groups (Control n = 338, 375, 160, 162, 170, 169, 151, 154, 182, 300; Y5R Ant n = 162, 146, 164, 145, 159, 122, 156, 220, 158, 300, 182, 270; in the order presented in the graph). The red lines represent the median; the black lines represent the quartiles. **d** Analysis of mean nuclear area for the SK-ES-1 xenografts shown in panel c (Control n = 10, Y5R Ant. n = 12 xenografts). Two-tailed unpaired t-test. **e** Primary tumor growth rate of SK-ES-1 xenografts, with or without Y5R antagonist treatment (n = 14 mice per group). Error bars indicate standard error of the mean.

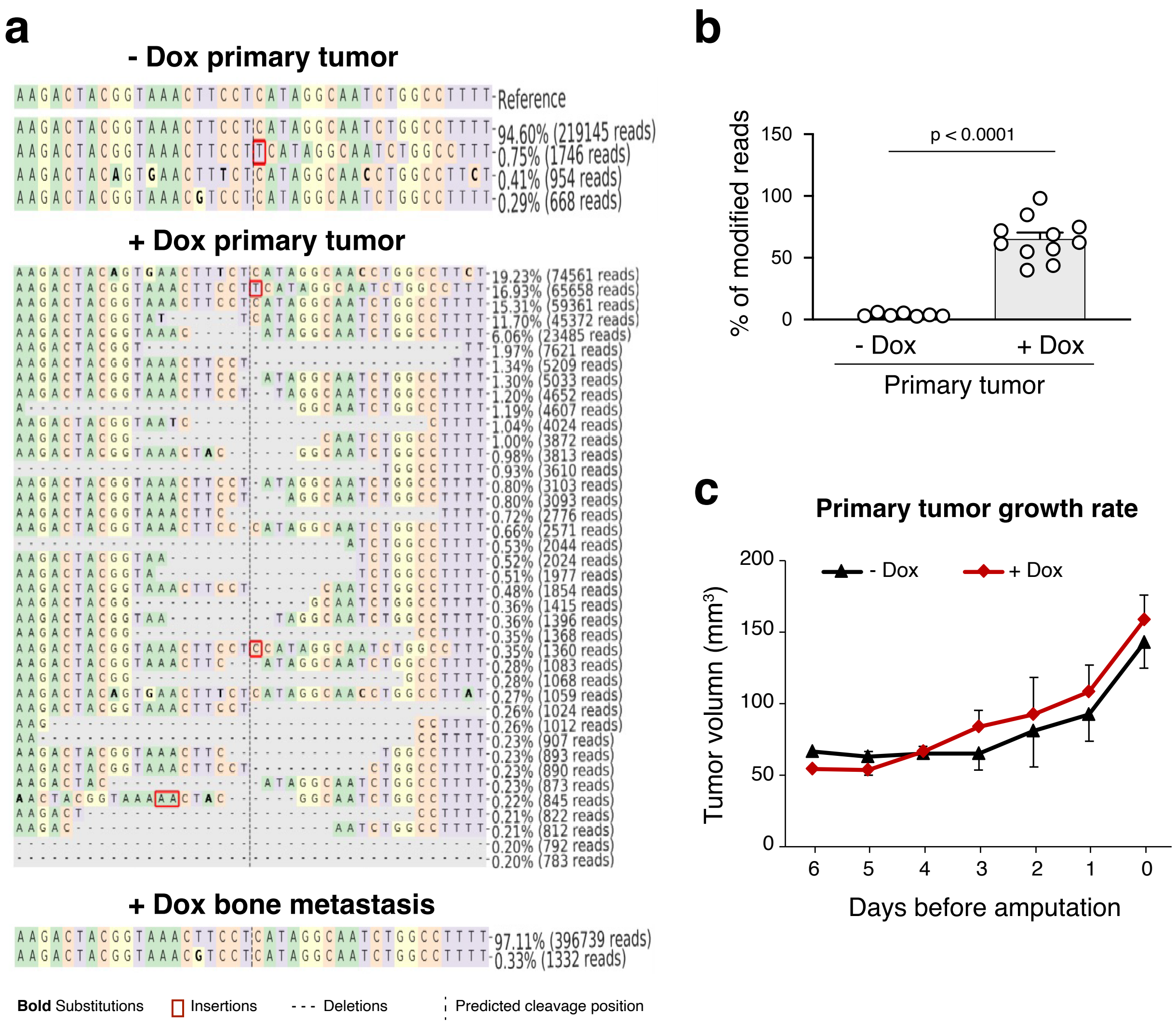

Supplementary figure 11. **Efficiency of *in vivo* NPY5R gene editing in SK-ES-1/Dox-Cas9/Y5R sgRNA xenografts.** **a** Representative *NPY5R* sequencing results of primary tumors with (+) and without (-) Dox treatment, as well as a distant bone metastasis from a mouse on +Dox diet. **b** Frequency of modified *NPY5R* sequencing reads in SK-ES-1/Dox-Cas9/Y5R sgRNA xenografts in mice on the control (-Dox) or doxycycline diet (+Dox) (n = 7, 11, respectively). Two-tailed unpaired t-test. **c** Primary tumor growth rate of SK-ES-1/Dox-Cas9/Y5R-sgRNA xenografts in mice on the control (-Dox) or doxycycline (+Dox) diet (n = 9 per group). Error bars indicate standard error of the mean.

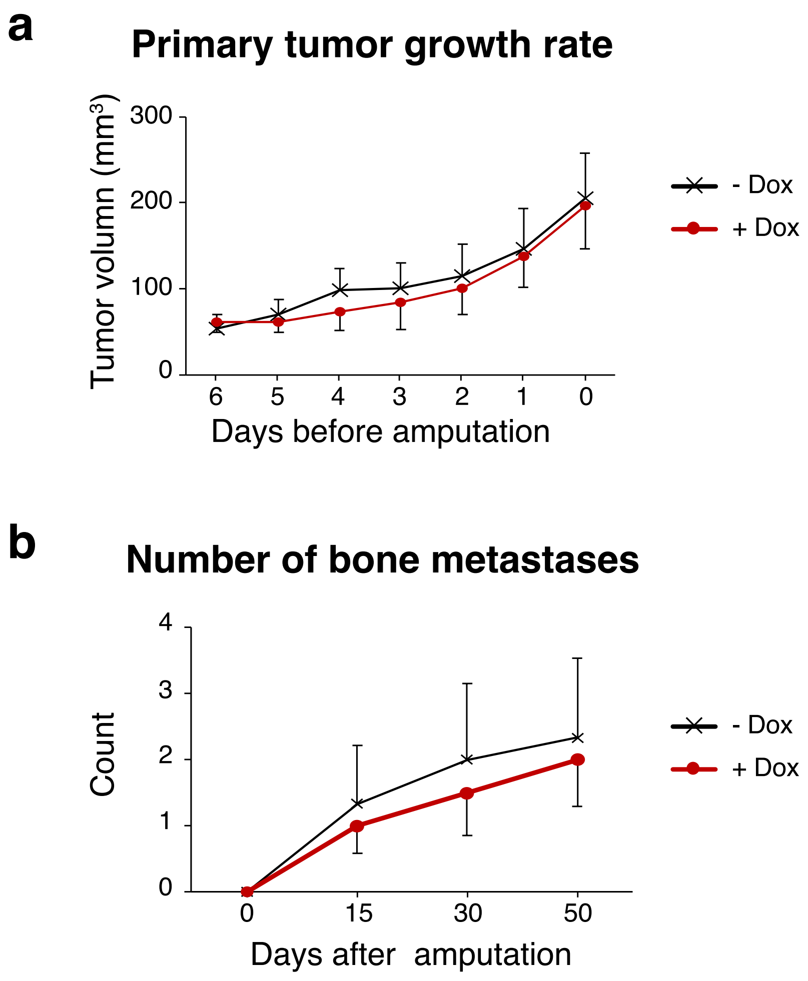

Supplementary figure 12. **Doxycycline does not affect primary tumor growth or metastasis rates in wild type (WT) SK-ES-1 xenografts. a** Primary tumor growth rate in mice bearing WT SK-ES-1 xenografts (n = 4 per group) on the control (-Dox) or doxycycline diet (+Dox). **b** Rate of bone metastasis formation in the above mice measured by MRI and confirmed by histopathological analysis. Error bars indicate standard error of the mean.

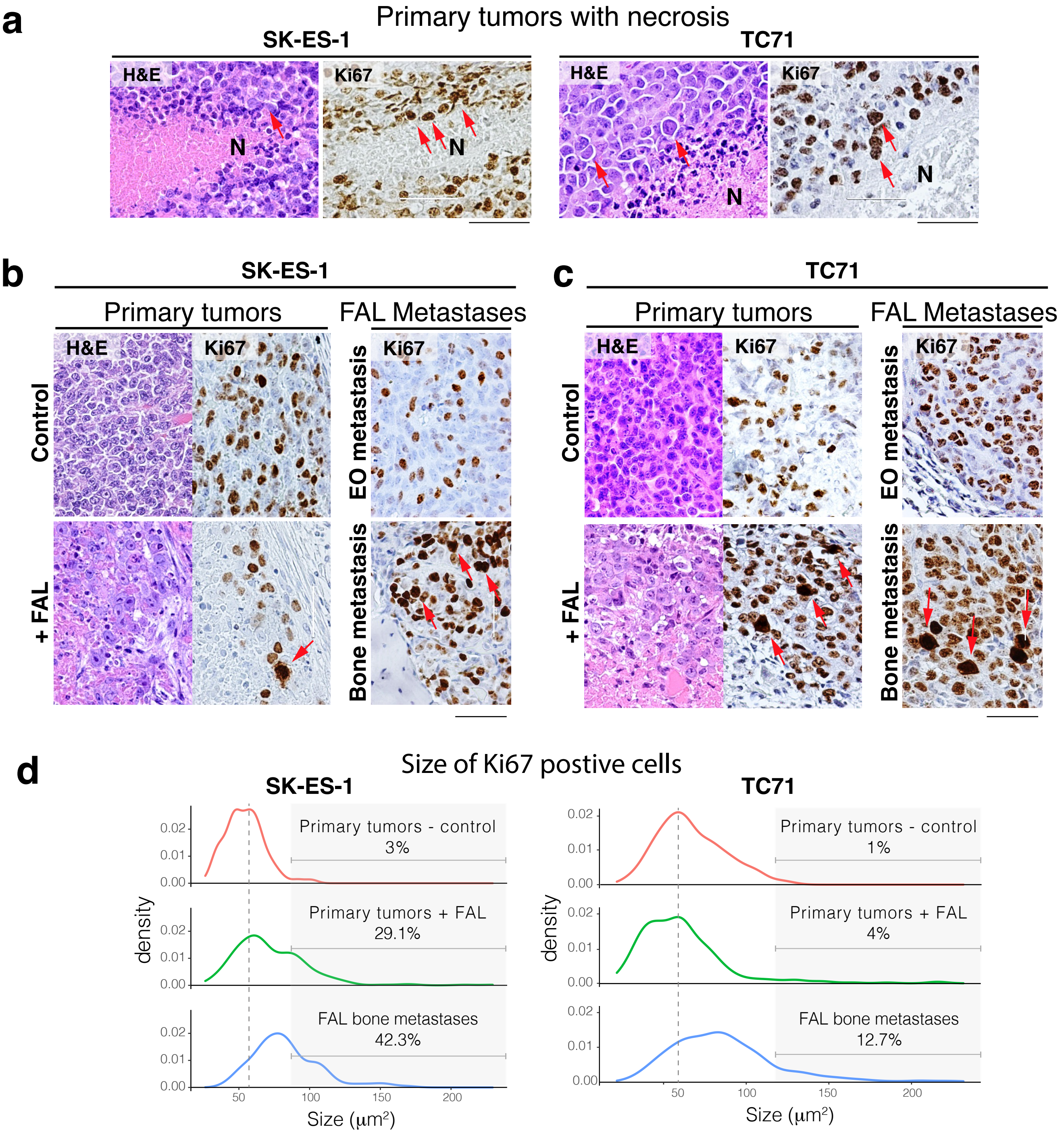

Supplementary figure 13. **Hypertrophic, proliferative ES cells accumulate in hypoxic areas of the primary tumors and hypoxia-induced bone metastases. a-c** Representative images of ES xenograft tissues stained with H&E or immunostained for Ki67. **a** Large SK-ES-1 and TC71 primary tumors (1000 mm^3) with hypertrophic, Ki67-positive cells at the edge of the necrotic areas (n = 3). **b-c** Control and FAL-exposed primary tumors (150 mm^3) and their corresponding bone or extraosseous (EO) metastases (n = 3 per group). **d** Analysis of nuclear area of Ki67-positive cells in tissues from control and FAL primary tumors, as well as hypoxia-induced bone metastases, depicted in panels b-c (SK-ES-1: Control primary tumors n = 215, FAL primary tumors n = 251; FAL metastases n = 274 cells; TC71: Control primary tumors n = 201, FAL primary tumors n = 298; FAL metastases n = 426 cells). N – necrosis; red arrows – hypertrophic cells. Scale bars: 50µm.

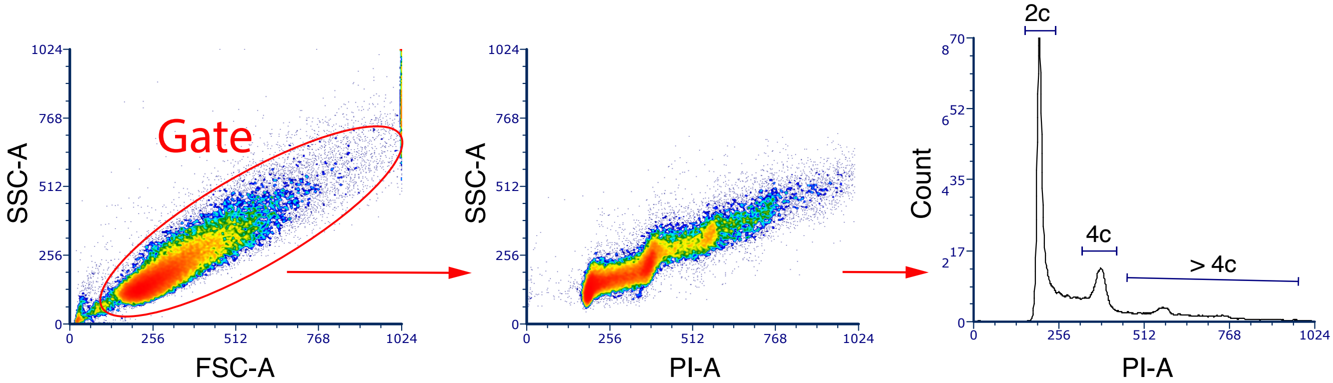

Supplementary figure 14. **Gating strategy for flow cytometry data analysis.**
