# Supplementary material for: Hypoxia-activated neuropeptide Y/Y5 receptor/RhoA pathway triggers chromosomal instability and bone metastasis in Ewing sarcoma
Source: Nat Commun. 2022 Apr 28;13:2323. doi: 10.1038/s41467-022-29898-x (PMC9051212; doi:10.1038/s41467-022-29898-x)
Supplement: Supplementary file 3 — Description of Additional Supplementary Information [file 41467_2022_29898_MOESM3_ESM.pdf]

**Description of Additional Supplementary Information**

1. **Supplementary figures 1-14**
2. **Supplementary movie 1:** Normal cell division in CHO-K1 cells transfected with Y1R-EGFP.
3. **Supplementary movie 2:** CHO-K1/Y5R-EGFP transient transfectants – cytokinesis failure leading to cell death.
4. **Supplementary movie 3:** CHO-K1/Y5R-EGFP transient transfectants – cytokinesis failure leading to the formation of polyploid cells.
5. **Source data**
