# Supplementary material for: Hypoxia-activated neuropeptide Y/Y5 receptor/RhoA pathway triggers chromosomal instability and bone metastasis in Ewing sarcoma
Source: Nat Commun. 2022 Apr 28;13:2323. doi: 10.1038/s41467-022-29898-x (PMC9051212; doi:10.1038/s41467-022-29898-x)
Supplement: Supplementary file 7 — Reporting Summary [file 41467_2022_29898_MOESM7_ESM.pdf]

Corresponding author(s): Joanna Kitlinska

Last updated by author(s): 03/09/2022

Reporting Summary

Nature Research wishes to improve the reproducibility of the work that we publish. This form provides structure for consistency and transparency in reporting. For further information on Nature Research policies, see our Editorial Policies and the Editorial Policy Checklist.

Statistics

For all statistical analyses, confirm that the following items are present in the figure legend, table legend, main text, or Methods section.

n/a	Confirmed	
☐	☒	The exact sample size (*n*) for each experimental group/condition, given as a discrete number and unit of measurement
☐	☒	A statement on whether measurements were taken from distinct samples or whether the same sample was measured repeatedly
☐	☒	The statistical test(s) used AND whether they are one- or two-sided<br>*Only common tests should be described solely by name; describe more complex techniques in the Methods section.*
☒	☐	A description of all covariates tested
☐	☒	A description of any assumptions or corrections, such as tests of normality and adjustment for multiple comparisons
☐	☒	A full description of the statistical parameters including central tendency (e.g. means) or other basic estimates (e.g. regression coefficient) AND variation (e.g. standard deviation) or associated estimates of uncertainty (e.g. confidence intervals)
☐	☒	For null hypothesis testing, the test statistic (e.g. *F*, *t*, *r*) with confidence intervals, effect sizes, degrees of freedom and *P* value noted<br>*Give P values as exact values whenever suitable.*
☒	☐	For Bayesian analysis, information on the choice of priors and Markov chain Monte Carlo settings
☒	☐	For hierarchical and complex designs, identification of the appropriate level for tests and full reporting of outcomes
☒	☐	Estimates of effect sizes (e.g. Cohen's *d*, Pearson's *r*), indicating how they were calculated

Our web collection on statistics for biologists contains articles on many of the points above.

Software and code

Policy information about availability of computer code

Data collection	No custom code was used for data collection.
Data analysis	FlowJo (Version 10.7.1) for FACS analysis. Image J (Version 1.51) and MetaMorph (Version 7.0) for image analysis. The data processing and statistical analysis was done using Prism 9, SAS 9.4, and RStudio (Version 1.4.1717). Analysis of CRISPR/Cas9 editing was performed using Cas-OFFinder and CRISPResso2.

For manuscripts utilizing custom algorithms or software that are central to the research but not yet described in published literature, software must be made available to editors and reviewers. We strongly encourage code deposition in a community repository (e.g. GitHub). See the Nature Research guidelines for submitting code & software for further information.

Data

Policy information about availability of data

All manuscripts must include a data availability statement. This statement should provide the following information, where applicable:

- Accession codes, unique identifiers, or web links for publicly available datasets
- A list of figures that have associated raw data
- A description of any restrictions on data availability

The authors declare that the data supporting the findings of this study are available within the paper and its supplementary information files.

Field-specific reporting

Please select the one below that is the best fit for your research. If you are not sure, read the appropriate sections before making your selection.

☒ Life sciences ☐ Behavioural & social sciences ☐ Ecological, evolutionary & environmental sciences

For a reference copy of the document with all sections, see nature.com/documents/nr-reporting-summary-flat.pdf

Life sciences study design

All studies must disclose on these points even when the disclosure is negative.

Sample size	For the animal experiments, the calculated sample size was 30 (15 mice in each group) based on the following calculations:<br>For the control group, the observed distant metastasis rate was 92% in the control group in our preliminary data. Assuming an alpha level of 5%, the sample size of 30 will achieve 82% power in the design with 3 repeated measures (MRI) having a AR(1) covariance structure if the distant metastasis rate is 60% with a one-sided test. The correlation between observations on the same subject is assumed to be 0.6. It would achieve about 90% power if the distant metastasis rate is 55% with all other assumptions the same as above. The same animal number will be used for other xenografts.<br>For the experiments on cellular models, no sample-size calculations were performed. The sample-size for each experiment were determined based on our previous data and similar published studies, as previously described (Gu et al., Methods Mol Biol., PMID: 21318814, Czarnecka et. Al, Neuropeptides 2019, PMID: 30503694, Abualsaud et al., Front Cell Dev Biol., 2021, PMID: 33681186). Each experiment was repeated at least two times.
Data exclusions	In the animal experiments, the mice that did not develop primary tumors or developed it with a significant delay as compared to the majority of the animals were excluded from analyses.
Replication	All cellular experiments have been conducted in at least two replicate experiments. Animal experiments were performed twice. All attempts at replication were successful.
Randomization	In animal experiments, mice were randomized into the experimental groups. For the experiments on cellular models, independent cell culture plates were assigned randomly to the experimental groups.
Blinding	All results presented here are purely based on objective analysis of the captured data, without possibility of subjective interpretation. Hence, blinding is not relevant to this study.

Reporting for specific materials, systems and methods

We require information from authors about some types of materials, experimental systems and methods used in many studies. Here, indicate whether each material, system or method listed is relevant to your study. If you are not sure if a list item applies to your research, read the appropriate section before selecting a response.

Materials & experimental systems

n/a	Involved in the study
☐	☒ Antibodies
☐	☒ Eukaryotic cell lines
☒	☐ Palaeontology and archaeology
☐	☒ Animals and other organisms
☒	☐ Human research participants
☒	☐ Clinical data
☒	☐ Dual use research of concern

Methods

n/a	Involved in the study
☒	☐ ChIP-seq
☐	☒ Flow cytometry
☒	☐ MRI-based neuroimaging

Antibodies

Antibodies used	Immunohistochemistry: Mouse monoclonal anti-CD99 (Clone 12 E7, IR057, DAKO; 1:50) and anti-CD68 (Clone KP1, GA609, DAKO; ready to use) antibody; rabbit polyclonal anti-pimonidazole (HypoxyprobeTM-1 kit; HPI, Inc.; 1:300), anti-CAIX (ab15086, Abcam; 1:1000), anti-Y5R (NB1-00957, Novus Biologicals; 1:300) and anti-Ki67 (ab15580, Abcam; 1:100) antibody; EnVision+ Single Reagents, HRP. Rabbit (K400311-2, DAKO; ready to use); EnVision+/HRP, Mouse, HRP. Mouse (K400111-2, DAKO; ready to use).<br><br>Immunocytochemistry: rabbit monoclonal anti-Y5R antibody (ab133757; Abcam; 1:250); mouse monoclonal anti-RhoA-GTP antibody (NE-26904; NewEast Biosciences; 1:100); AlexaFluor 594-conjugated donkey anti-mouse antibody (A-21203, Invitrogen; 1:1000); Alexa Fluor 488-conjugated goat anti-rabbit antibody (A-11008, Invitrogen; 1:1000).<br><br>Western blot: Mouse monoclonal anti-alpha 1 sodium-potassium ATPase (ab7671, Abcam; 1:1000), phospho-p44/42 MAPK (Erk1/2) (Thr202/Tyr204) (E10) (9106, Cell Signaling Technologies; 1:2000), anti-RhoA IgM (ARH05, Cytoskeleton Inc.; 1:500) and anti-β-actin antibody (A1978, Sigma; 1:10000); goat polyclonal anti-Y5R antibody (EB06769; Everest Biotech; 1:1000); rabbit polyclonal anti-

	p44/42 MAPK (Erk1/2) Antibody (9102, Cell Signaling Technologies; 1:2000); ECL Rabbit IgG, HRP-linked F(ab')$_2$ fragment (from donkey) (NA9340-1ML, Amersham; 1:1000); Mouse IgG HRP Linked Whole Ab (NA931-1ML, Amersham; 1:1000); mouse anti-goat IgG-HRP (sc-2354, Santa Cruz; 1:1000).
Validation	All antibodies were commercially available and validated by the companies for the target specificity and species cross reactivity. For detailed information please refer to the manufacturers' websites. In addition, the validation under specific assay conditions used in this study was performed based on the molecular weight of the band and the use of appropriate positive or negative controls (Western blot) or sub-cellular localization, cell morphology and the use of internal and external positive and negative control tissues (immunostaining in cells and tissues). The anti-Y5R antibody were validated in cells and tissues expressing subjected to CRISPR/Cas9 NPY5R gene knockout.

Eukaryotic cell lines

Policy information about cell lines

Cell line source(s)	SK-N-MC, SK-N-BE(2), CHO-K1 - American Type Culture Collection (ATCC, Manassas, VA); SK-ES-1, TC71, TC32, A4537, ES925, 5838, RDES, MHH-ES-1 – Dr. Jeffrey Toretsky, Georgetown University
Authentication	The cell lines were authenticated by genetic fingerprinting at the Georgetown University Medical Center Tissue Culture Shared Resources. The cell lines were authenticated before the initiation of in vitro and in vivo experiments.
Mycoplasma contamination	The cells were routinely tested for mycoplasma contamination using the MycoAlert™ PLUS Mycoplasma Detection Kit (Lonza). Only cells negative for mycoplasma were used for the experiments.
Commonly misidentified lines (See ICLAC register)	One of the cell lines used, SK-N-MC, is commonly misidentified as a neuroblastoma cell line, while its actual origin is a Ewing sarcoma tumor. In this study, we used this cell line correctly, as a Ewing sarcoma cell line (Supplementary figure 7).

Animals and other organisms

Policy information about studies involving animals; ARRIVE guidelines recommended for reporting animal research

Laboratory animals	Fox Chase SCID® Beige mice (CB17.Cg-PrkdcscidLystbg/Crl Strain Code: 250), Charles River Laboratories; 3-4 weeks old female. TH-MYCN mice - 129X1/SvJ-Tg(TH-MYCN)41Waw/Nci; Frederic National Laboratory; NCI Mouse Repository; 6-12 weeks old males and females.
Wild animals	No wild animals were used in the study.
Field-collected samples	No field collected samples were used in the study.
Ethics oversight	Georgetown University Institutional Animal Care and Use Committee.

Note that full information on the approval of the study protocol must also be provided in the manuscript.

Flow Cytometry

Plots

Confirm that:

☒ The axis labels state the marker and fluorochrome used (e.g. CD4-FITC).

☒ The axis scales are clearly visible. Include numbers along axes only for bottom left plot of group (a 'group' is an analysis of identical markers).

☒ All plots are contour plots with outliers or pseudocolor plots.

☒ A numerical value for number of cells or percentage (with statistics) is provided.

Methodology

Sample preparation	Cells were fixed in 75% ethanol, and stained with propidium iodide (PI) according to the standard procedures.
Instrument	Flow cytometry data acquisition and sorting were performed on LSR-Fortessa (Becton Dickinson, Franklin Lakes, NJ).
Software	FlowJo (Threestar) was used for flow cytometry data analysis.
Cell population abundance	For all flow cytometry data acquisition, at least 5x10^6 cells were sorted. Propidium Iodide staining was performed and used for DNA content analysis.
Gating strategy	Cells were gated were gated by forward and side scatter to remove debris and potential multiplets.

☒ Tick this box to confirm that a figure exemplifying the gating strategy is provided in the Supplementary Information.
